# Modifications of the endosomal compartment in fibroblasts from sporadic Alzheimer’s disease patients are associated with cognitive impairment

**DOI:** 10.1038/s41398-023-02355-z

**Published:** 2023-02-14

**Authors:** Laura Xicota, Julien Lagarde, Fanny Eysert, Benjamin Grenier-Boley, Isabelle Rivals, Alexandra Botté, Sylvie Forlani, Sophie Landron, Clément Gautier, Cecilia Gabriel, Michel Bottlaender, Jean-Charles Lambert, Mounia Chami, Marie Sarazin, Marie-Claude Potier

**Affiliations:** 1 ICM Paris Brain Institute, CNRS UMR7225, INSERM U1127, Sorbonne University, Hôpital de la Pitié-Salpêtrière, 47 Bd de l’Hôpital, 75013 Paris, France; 2 Department of Neurology of Memory and Language, GHU Paris Psychiatrie & Neurosciences, Hôpital Sainte Anne, F-75014 Paris, France; 3 Université Paris Cité, F-75006 Paris, France; 4 Université Paris-Saclay, BioMaps, Service Hospitalier Frederic Joliot CEA, CNRS, Inserm, F-91401 Orsay, France; 5 Institut of Molecular and Cellular Pharmacology, Laboratory of Excellence DistALZ, Université Côte d’Azur, INSERM, CNRS, Sophia-Antipolis, F-06560 Valbonne, France; 6 Univ. Lille, Inserm, CHU Lille, Institut Pasteur de Lille, U1167-RIDAGE– Facteurs de risque et déterminants moléculaires des maladies liées au vieillissement, F-59000 Lille, France; 7 Equipe de Statistique Appliquée, ESPCI Paris, INSERM, UMRS 1158 Neurophysiologie Respiratoire Expérimentale et Clinique, PSL Research University, Paris, France; 8 ICM DNA and Cell Bank CNRS UMR7225, INSERM U1127, Sorbonne University, Hôpital de la Pitié-Salpêtrière, 47 Bd de l’Hôpital, 75013 Paris, France; 9 Institut de Recherche Servier, 125 Chem. de Ronde, 78290 Croissy sur Seine, France; 10 CEA, Neurospin, UNIACT, Paris Saclay University, 91191 Gif-sur-Yvette Cedex, France

**Keywords:** Diagnostic markers, Molecular neuroscience

## Abstract

Morphological alterations of the endosomal compartment have been widely described in post-mortem brains from Alzheimer’s disease (AD) patients and subjects with Down syndrome (DS) who are at high risk for AD. Immunostaining with antibodies against endosomal markers such as Early Endosome Antigen 1 (EEA1) revealed increased size of EEA1-positive puncta. In DS, peripheral cells such as peripheral blood mononuclear cells (PBMCs) and fibroblasts, share similar phenotype even in the absence of AD. We previously found that PBMCs from AD patients have larger EEA1-positive puncta, correlating with brain amyloid load. Here we analysed the endosomal compartment of fibroblasts from a very well characterised cohort of AD patients (IMABio3) who underwent thorough clinical, imaging and biomarkers assessments. Twenty-one subjects were included (7 AD with mild cognitive impairment (AD-MCI), 7 AD with dementia (AD-D) and 7 controls) who had amyloid-PET at baseline (PiB) and neuropsychological tests at baseline and close to skin biopsy. Fibroblasts isolated from skin biopsies were immunostained with anti-EEA1 antibody and imaged using a spinning disk microscope. Endosomal compartment ultrastructure was also analysed by electron microscopy. All fibroblast lines were genotyped and their AD risk factors identified. Our results show a trend to an increased EEA1-positive puncta volume in fibroblasts from AD-D as compared to controls (p.adj = 0.12) and reveal enhanced endosome area in fibroblasts from AD-MCI and AD-AD versus controls. Larger puncta size correlated with PiB retention in different brain areas and with worse cognitive scores at the time of biopsy as well as faster decline from baseline to the time of biopsy. Finally, we identified three genetic risk factors for AD (*ABCA1*, *COX7C* and *MYO15A*) that were associated with larger EEA1 puncta volume. In conclusion, the endosomal compartment in fibroblasts could be used as cellular peripheral biomarker for both amyloid deposition and cognitive decline in AD patients.

## Introduction

Alzheimer’s disease (AD) is the most common cause of dementia and represents a major public health concern, with >130 million cases worldwide anticipated by 2050. The key proteins linked to the pathophysiology of AD are the amyloid precursor protein (APP) and tau. APP can be metabolised through two main pathways, the non-amyloidogenic and the amyloidogenic pathway. It is the latter, APP that gives rise to the toxic Aβ peptides that will aggregate in AD [1]. The conditions needed for the amyloidogenic cleavage of APP are found in the endo-lysosomal pathway, and it has been demonstrated that Aβ production is dependent on the internalisation of the APP protein [2]. In addition, hyperphosphorylated tau has also been proven to interact with endocytic proteins leading to dysregulation of key components of this pathway [3]. Alterations of the endo-lysosomal compartment have been described in pyramidal neurons from *post-mortem* brains of AD patients, including increased number of lysosomes and enlarged puncta labelled with specific antibodies against the early endosomal protein Rab5 [4, 5]. Increased size of Rab5 positive or early endosome antigen 1 (EEA1) positive puncta, another early endosomal marker, have also been observed in pyramidal neurons in pre-AD and in Down syndrome (DS), before amyloid deposition [6, 7]. In addition, higher levels of Aβ in the frontal cortex of pre-AD brains have been linked to enlarged Rab5 positive puncta, and intracellular accumulations of Aβ peptides colocalize with Rab5 [7]. One of the main remaining questions is whether alterations of the endosomal compartment lead to increased amyloid production or the other way around. Overexpression of Rab5 induced an increase in Aβ production [8] and an increase in tau phosphorylation [9]. Likewise, increasing β-CTF, the first cleavage product of APP by the β-secretase BACE1 led to higher Rab5 activation [10]. Alterations of the endo-lysosomal compartment are not exclusive to AD as several genes involved in the pathology of Parkinson’s disease are linked to the endo-lysosomal pathways [11–13], and specific modifications of this pathway have been observed in Huntington’s disease [14], among others.

Alterations of the endo-lysosomal pathway are not only found in the nervous system but also in peripheral cells in AD and related diseases. Indeed, fibroblasts and peripheral blood mononuclear cells (PBMCs) from individuals with DS showed enlarged Rab5 and EEA1 positive puncta [15, 16]. Recently, we characterised the endosomal compartment of fibroblasts from individuals with DS and euploid controls using aldehyde fixation and high-pressure freezing electron microscopy. We found in the DS cells that enlarged EEA1 positive puncta seen under the confocal microscope localised on clusters of normal-sized endosomes likely caused by aldehyde fixation, while without aldehyde fixation endosomes appeared more numerous, non-clustered and with no significant size difference as compared to control cells [17]. In PBMCs from sporadic AD patients, we found an increase in the proportion of cells carrying larger EEA1 puncta. Of note, the enlarged volume of the puncta was significantly positively correlated to brain amyloid deposition, particularly in the precuneus [18].

Finally, alterations of endo-lysosomal markers (EEA1, Rab7, LAMP1, and LAMP2) have been observed in the cerebrospinal fluid (CSF) of patients with AD [19]. Interestingly, in the CSF, some of these proteins correlated with AD biomarkers such as LAMP2 and phospho-tau 181 or EEA1 and total-tau. However, a recent study by the same group using parallel reaction monitoring-mass spectrometry (PRM-MS) failed to confirm these results [20].

The objective of our study was to ascertain changes in the endosomal compartment in fibroblasts from a monocentric longitudinal and well characterised cohort of sporadic AD patients and controls (IMABio3) and correlate them with cognitive, neuroimaging features of the disease and genetic risk factors, thus evaluating their potential use as cellular biomarkers of AD.

## Materials and methods

### Participants

Subjects who had undergone a skin-punch biopsy were selected from the IMABio3 cohort (PHRC-0054-N 2010; NCT01775696). The study was approved by the Ethics Committee of the Salpêtrière Hospital. All subjects provided written informed consent prior to participating.

Patients with AD were included according to the following criteria: (i) progressive episodic memory impairment, characterised by a low free recall not normalised with semantic cueing [21, 22]; (ii) absence of extrapyramidal signs and (iii) CSF AD profile defined as score <0.8, calculated with the formula amyloid-β42/[240 + (1.18 *total-tau)] [23].

Controls were recruited according to the following criteria: (i) Mini-Mental State Examination (MMSE) score >27/30 and normal neuropsychological assessment; (ii) Clinical Dementia Rating (CDR) score = 0; (iii) no history of neurological or psychiatric disorders and (iv) no memory complaint or cognitive deficit.

All participants underwent the same procedure including a complete clinical and neuropsychological assessment, brain 3 T MRI, and ^11^C-PiB PET imaging, and were followed up annually for 2 years. Skin biopsies were performed one time at the 1-year or final (2-year) visit. CSF extraction was only performed for subjects that were not controls.

They were classified as Controls, Mild Cognitive Impairment (AD-MCI), or AD with profound cognitive decline/dementia (AD-D), according to their clinical status as measured by the Clinical Dementia Scale (CDR) and the Mini Mental State examination (MMSE).

### Regional brain amyloid burden assessment by PiB-PET

Amyloid burden was determined in several brain regions by PiB retention at baseline as previously described (Standard Uptake Value ratio, SUVr; [24]). In summary, parametric images were created using BrainVisa software (http://brainvisa.info) on averaged images over 40–60 min after injection of [^11^C]-PiB. Standard Uptake Value ratio (SUVr) parametric images were obtained by dividing each voxel by the corresponding value found in the cerebellar grey matter eroded (4 mm). We considered the following VOIs: frontal cortex, anterior cingulate, middle cingulate, posterior cingulate, precuneus, parietal cortex, temporal cortex, and the hippocampus. PiB Global Cortical Index (GCI), representing the subject’s mean SUVr of the neocortical regions was also determined. The cut-off value of PiB GCI positivity was set as 1.45.

### Cell culture

Punch biopsy samples of the skin were rinsed in 1 mL of culture medium (DMEM 1X, FBS 15%, Penicillin/Streptomycin 0.5%, Hepes 10 mM 1%, L-Glutamine 2 mM 1%) in a B6 petri dish and cut into smallest possible fragments using two scalpels. Three mL of culture medium were added and fragments were homogenised using of a sterile 5 mL pipette. Two mL of this suspension were transferred to 2 T25 culture flasks (1 mL per flask) and incubated at 37 °C, CO2 5%. After 48H, 0.5 mL of culture medium were added in each flask and at day 8–9 culture medium was replaced, then twice a week. When cells began to form dense foci and fibroblasts within these foci reached confluence, cells were transferred from T25 to T75 using trypsin (0.05% Trypsine/0.02% EDTA in PBS 5 min at 37 °C) in a final volume of 8 mL medium then changed two to three times a week. When confluent, fibroblasts were frozen at −80 °C in 90% SVF + 10% DMSO at a final concentration of around 1 million cells/mL.

Fibroblasts were maintained at low passages (<9) and cultured in T75 flasks in high glucose, GlutaMAX, pyruvate, DMEM media (GIBCO), supplemented with 10% FBS, and with 1% penicillin-streptavidin antibiotics. When confluent, cells were distributed into 96-well microtiter plates suitable for image acquisition (glass-bottom plates). A maximum of 5 cell lines were plated per plate, with three different densities (2500, 5000, and 10,000 cells plated per well) with four replicates per density. Cells were fixed in 4% PFA after 48 h or once the desired confluence was reached.

The choice of fibroblasts over PBMCs for analysis was done considering that fibroblasts have a larger cytoplasm, making it easier to characterise EEA1 puncta. Our previous studies showed that the number of EEA1 positive puncta was around 15–20 in PBMCs [18] while it was about 50 fold higher in fibroblasts [17]. Besides fibroblasts are easy to maintain in culture and proliferate without the need of external agents.

### Immunofluorescence

Cells were permeabilized with PBS 0.2% Triton before blocking with 3% BSA for 45 min. The primary antibody was anti-EEA1 to label early endosomes (#3288, Cell Signalling). Cells were incubated with the primary antibody (1:500) for 1 h at room temperature, followed by 1 h incubation with the secondary antibody tagged with a far-red fluorescent molecule (A-21245, 1:1000) and Phalloidin (green). After three five-minute rinses in PBS 1X cells were stained with DAPI (blue). Images were taken on an iMIC TILL Photonics inverted microscope using an oil immersion 63X objective, 7*7 mosaic images were taken, with a z step of 0.25 µm, encompassing the whole volume of the cells. Regions to be imaged were manually selected after observing the well at a lower magnification to maximise the number of cells in the imaging field. Image analysis was performed with the ICY software on 3D stacks (https://icy.bioimageanalysis.org). To do so, we manually drew the contours of the cells based on the Phalloidin staining and used the Spot detector plug-in to analyse the puncta, with a minimum size of three voxels. A total of 75 cells for each subject were analysed and included in the data. The investigator was blinded to group while performing image analysis.

### Electron microscopy

Human fibroblasts were fixed in 1.6% glutaraldehyde in 0.1 M phosphate buffer (pH 7.4), rinsed with cacodylate buffer 0.1 M, and then post-fixed in osmium tetroxide (1% in cacodylate buffer) reduced with potassium ferrycyanide (1%) for 1 h. Cells were dehydrated with several incubations in increasing concentrations of ethanol or acetone, respectively, and embedded in epoxy resin (EPON), and 70 nm ultrathin sections were contrasted with uranyl acetate and lead citrate and observed with a Transmission Electron Microscope (JEOL JEM 1400) operating at 100 kV and equipped with a Olympus SIS MORADA camera. We used ImageJ software to quantify endosomes area.

### Genotyping and imputation

Samples from the IMABio3 cohort were genotyped with the Illumina Infinium Global Screening Array (GSA, GSAsharedCUSTOM_24 + v1.0) and the calling was generated by the “Centre National de Recherche en Génomique Humaine” (Evry, France). The quality control (QC) followed the same steps performed in the European Alzheimer & Dementia Biobank (EADB) dataset and described here [25]. Using the same variants included in the main EADB batch sample QC, samples with missingness >0.05, sex inconsistencies or with an heterozygosity rate that deviates >± 6 standard deviations from the mean computed in the main EADB batch, were excluded. To identify population outliers, the samples were projected onto a Principal Component Analysis using the 1000 Genomes Phase3 population [26] as the reference. Samples falling outside the interval median ± 12 median absolute deviation computed in the main EADB batch on any of the first two principal components were flagged as outliers. To control for cryptic relatedness, we excluded one individual from each pair of samples with a kinship coefficient more than 0.046875 (cut-off for second-degree relatives). Then, we performed the variant QC on the genotyping batch of the IMABio3 cohort, restricted to the list of variants passing all steps in the variant QC in the main EADB batch. We excluded variants showing a missingness >0.05 or having a *P* value of the Fisher’s exact test on cases/controls missing calls <1e−10 or a *P* value of the Hardy-Weinberg equilibrium test performed in controls <5e−8. The remaining variants were then assessed by comparing their frequencies against two reference panels (i.e. the Haplotype Reference Consortium r1.1 (HRC) [27] excluding 1000 Genomes samples and the Genome Aggregation Database v3 (gnomAD) non-Finnish European samples [28]. Allele counts were then compared to the IMABio3 genotyping batch counts by performing a chi-square test (χ2); variants showing a χ2 > 500 in both HRC and gnomAD, or a χ2 > 500 in one reference panel and not present in the other, were excluded. All samples and variants passing quality control were then imputed with the Trans-Omics for Precision Medicine (TOPMed) Freeze 5 reference panel [29] on the Michigan Imputation Server [30] Statistical analysis.

### Data analyses

Data were processed and analysed using R 4.0.3 [31]. ANOVA or Kruskal–Wallis analysis were performed at the subject level, as appropriate. The plate effect was assessed as a random effect, both directly and as an interaction with the group in a linear model. Post-hoc pairwise tests were performed using Tukey and Dunn test respectively. Correlations between pairs of variables were evaluated using Pearson’s linear correlation coefficient. Association with genetic variants was performed using ANOVA with post-hoc Tukey tests, due to the sample size, only AD status was included as covariate in the analysis.

## Results

### Demographic description

A total of 21 subjects (7 controls, 7 AD-MCI and 7 AD-D) who had undergone skin biopsies were selected from the IMABio3 cohort. The full demographic description is detailed on Table 1. Subjects from the control group were older at the time of biopsy, but not significantly as compared to the two other groups. In terms of sex distribution, only women were available in the AD-D group, therefore we could not match the groups by sex. Similarly, because of the high prevalence of the *APOEε4* allele in AD, the distribution of *APOEε4* carriers was not equal through diagnostic groups, with none of the *ε4* carriers in the control group. As expected, PiB GCI values were higher in AD-D compared to controls (*p* = 0.0016), and AD-MCI (*p* = 0.0049) compared to controls, but no difference could be observed between the two AD groups. Both CDR sum of boxes (SOB) and MMSE were categorising criteria for this study, therefore AD-D had significantly higher CDR SOB and lower MMSE than either AD-MCI (*p* < 0.0001 and *p* = 0.0008) or controls (*p* < 0.0001 and *p* < 0.0001), as expected, AD-MCI had higher CDR SOB (*p* = 0.0004) and lower MMSE (*p* = 0.04) than controls.

**Table 1:** Demographic description of the sample according to diagnostic group.

		Controls (*n* = 7)	AD-MCI (*n* = 7)	AD-D (*n* = 7)
Age	Mean ± SD	71 ± 12.3	63.8 ± 9.3	64.4 ± 10.3
	Range	53–88.6	53.7–81	53.9–82.7
Sex	Female	3 (43%)	3 (43%)	7 (100%)
	Male	4 (57%)	4 (57%)	0 (0%)
APOE	E3/E3	7 (100%)	1 (14%)	1 (14%)
	E3/E4	0 (0%)	5 (72%)	3 (43%)
	E4/E4	0 (0%)	1 (14%)	3 (43%)
PiB GCI	Median	1.42 (1.19–3.1)	2.87 (2.38–3.44)	2.94 (2.37–4.16)
CDR SOB (/18)	Median	0.0 (0.0–1.0)	4.5 (3.0–6.0)	11.0 (7.0–15.0)
MMSE (/30)	Median	29.0 (25.0–30.0)	23.0 (14.0–27.0)	8.0 (5.0–23.0)
CSF tau (pg/ml)	Mean	NA	651.4 ± 291.5	591.6 ± 283.3
	Range	NA	349–1200	265–947
CSF ptau (pg/ml)	Mean	NA	82.57 ± 22.6	71.29 ± 26.3
	Range	NA	55–119	38–104
Ab 42 CSF (pg/ml)	Mean	NA	437.9 ± 98.6	433.1 ± 86.9
	Range	NA	299–538	323–558

### Early endosomal compartment characterisation

**Fig. 1:**
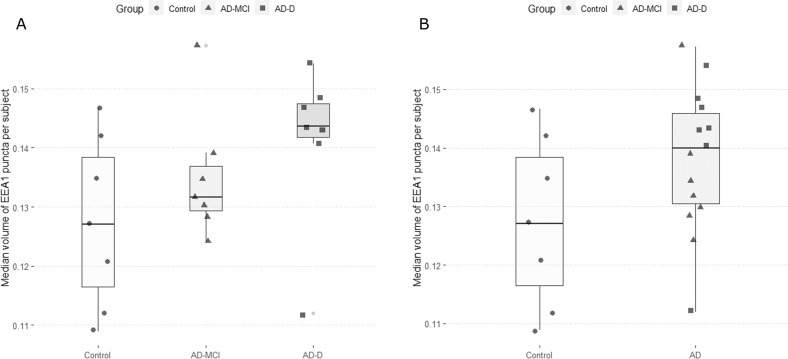
EEA1 positive puncta volumes in fibroblasts of controls, AD-MCI and AD-D. **A** Controls are represented as circles and their quartile distribution as a white-colored boxplot, AD-MCI are represented as triangles and AD-D as squares, their respective quartile boxplots are in light and dark grey. **B** Pooled AD subjects are represented as triangles for AD-MCI and squares for AD-D and their pooled quartile distribution is represented in light grey.

We analysed a total of 75 cells per subject to obtain median EEA1 puncta volume per cell. This median volume per cell was then used to calculate the median EEA1 positive puncta volume per subject. Differences were investigated between controls and the AD groups (AD-MCI and AD-D) taken together or separately. Uncorrected Kruskal–Wallis Dunn test post-hoc analysis showed a trend for a difference between AD-D and controls (Fig. 1A) (p.adj = 0.12). The pooled analysis showed a trend for higher values in the AD group (Fig. 1B) (p.adj = 0.108).

**Fig. 2:**
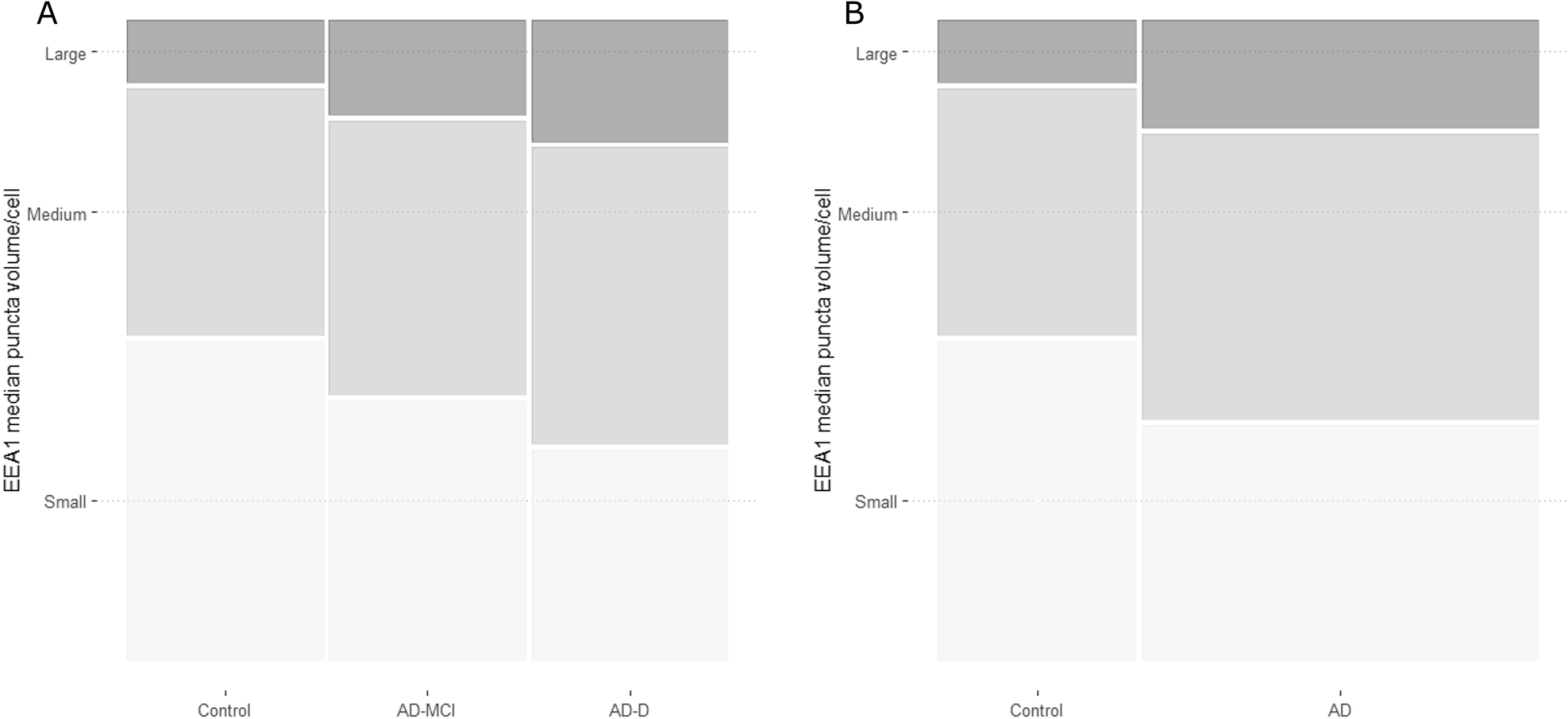
Cell distribution according to their categorical median EEA1 puncta volume. The proportion of cells with a median EEA1 puncta volume ≤50th centile of the control group (small) is represented in light grey, the proportion of cells between the 50th and the 90th centile of the control group (medium) is represented in grey, and the proportion of cells ≥90th centile (large) is represented in dark grey. Panel **A** represents the groups according to diagnostic group, and panel **B** represents them according to disease group.

Cells were categorised according to their median EEA1 puncta volume as cells with small, medium, or large puncta. To do this categorisation we calculated the 50th and 90th centile of the distribution of puncta volume obtained with controls. Cells that had a median puncta volume below the 50th centile were classified as having small median puncta volume, cells that fell between the 50th centile and the 90th were classified as having medium median puncta volume, and the rest were classified as having large puncta volume. Subsequently, the proportions of cells from each class were analysed. Both AD-MCI and AD-D had significantly different proportions of the three classes compared to controls (*p* < 0.0001) and the difference between AD groups was also significant (*p* < 0.0001) (Fig. 2A). These different proportions relied on a lower percentage of cells with small EEA1 positive puncta volume (≤50th centile) and an increase of cells with both medium (>50th < 90th centiles) and large (≥90th centile) EEA1 positive puncta volume. When pooling together the two AD groups, differences in the proportions with control cells remained significant (*p* < 0.0001) (Fig. 2B).

**Fig. 3:**
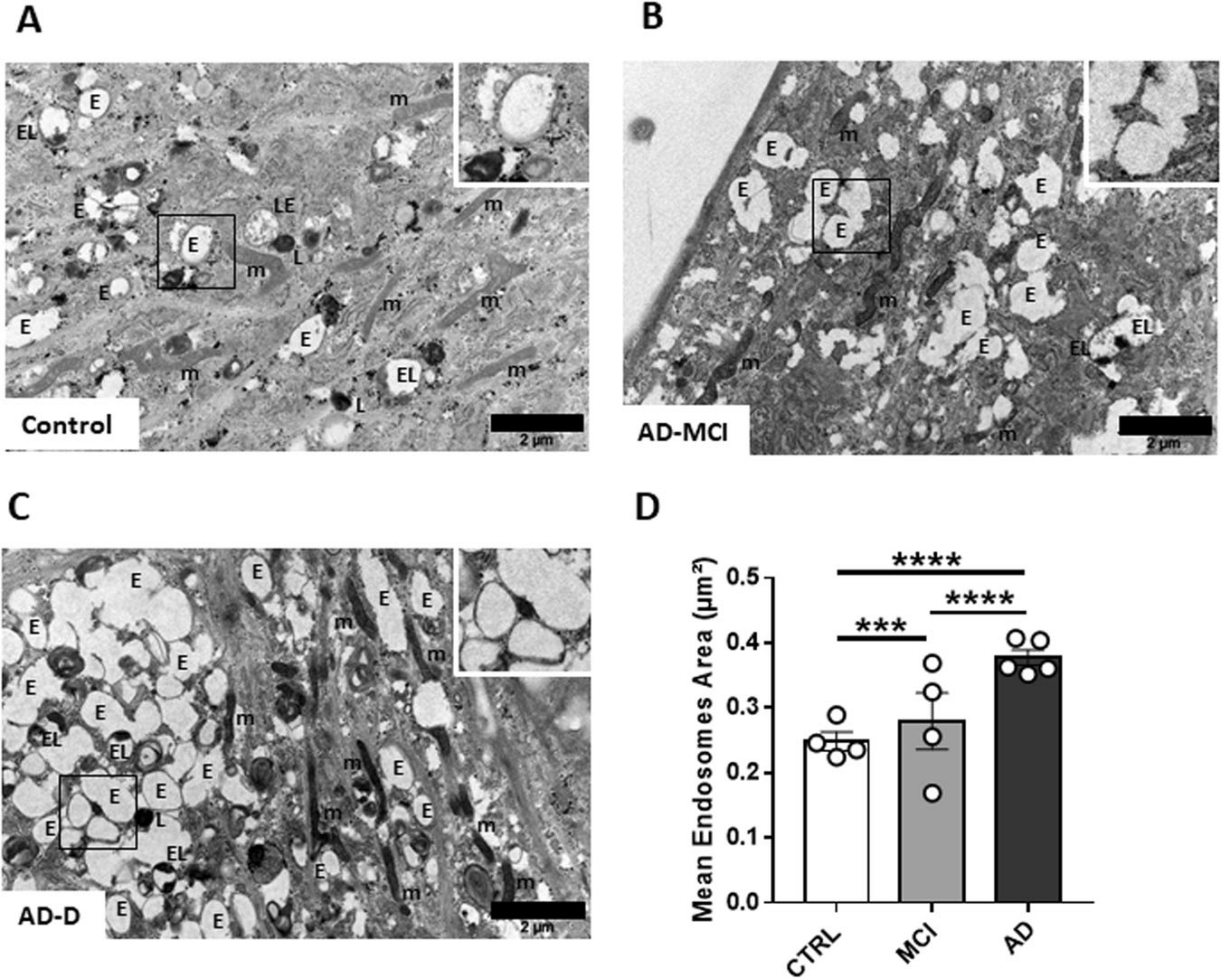
Ultrastructural imaging of the endosomal compartment in fibroblasts of controls. **A** AD-MCI (**B**) and AD-D (**C**). The squared zones are magnified at the upper right of each image. **D** Analysis of the mean endosome area (µm^2^) on at least 20 different fields (>200 endosomes) obtained from controls (*n* = 3), AD-MCI (*n* = 4) and AD-D (*n* = 4). m mitochondria, L Lysosome, E Endosome, EL Endolysosome, and LE Late Endosome.

We next explored the endosomal ultrastructure in fibroblasts from controls, AD-MCI and AD-D using electron microscopy. Figure 3A showing representative images of fibroblasts from each group revealed enlarged endosomal compartment in AD groups versus controls. The quantitative analyses demonstrated a significant increase of the mean endosomal area between AD groups and controls as well as between AD-D and AD-MCI (Fig. 3B) thus confirming changes of the endosomal compartment on AD condition.

### Correlations between EEA1 positive puncta volume and PiB retention

**Fig. 4:**
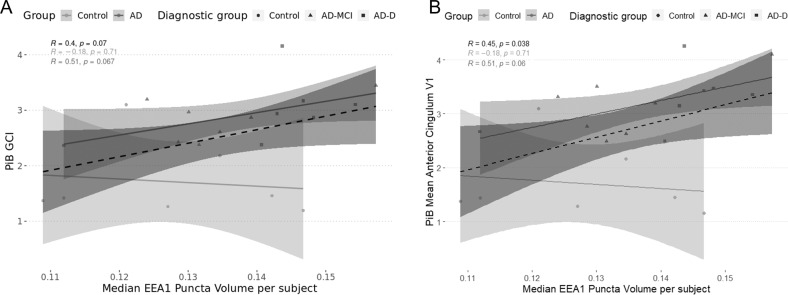
Graphical representations of the correlations between PiB retention and the median EEA1 positive puncta volume. For all cases, circles represent controls, triangles represent AD-MCI, and squares represent AD-D. Likewise, the total correlation is represented as a dashed line in dark-grey and its 95% confidence interval is represented in the shaded dark grey area. The stratified correlations according to disease group are in grey (AD) and light grey (Control). Correlation values are on the graph, with the total correlation being in black, the correlation for controls in light grey, and the correlations for AD in dark grey. **A** shows correlations between global PiB retention and EEA1 positive puncta volume, **B** presents correlations between PiB retention in the anterior cingulum and EEA1 positive puncta volume.

Previously, we had observed a correlation between EEA1 positive puncta size in PBMCs from AD patients and PiB accumulation in their brain [18]. For this study, we performed total correlations including all subjects independently of diagnostic group, and stratified correlations separating controls and AD. The reason for these stratified correlations is the floor effect for some of the regional SUVr observed in the control group. When performing the total correlations (Supplementary Table 1) only the one with PiB retention in the anterior cingulum was significant (Supplementary Table 1 in bold and Fig. 4B), with a non-significant trend for global PiB retention (Supplementary Table 1 in italics and Fig. 4A). However, none of the correlations remained significant after correction for multiple testing. For the control group, no significant correlations could be observed (Supplementary Table 1).

### Correlations of median puncta volume and cognition

**Fig. 5:**
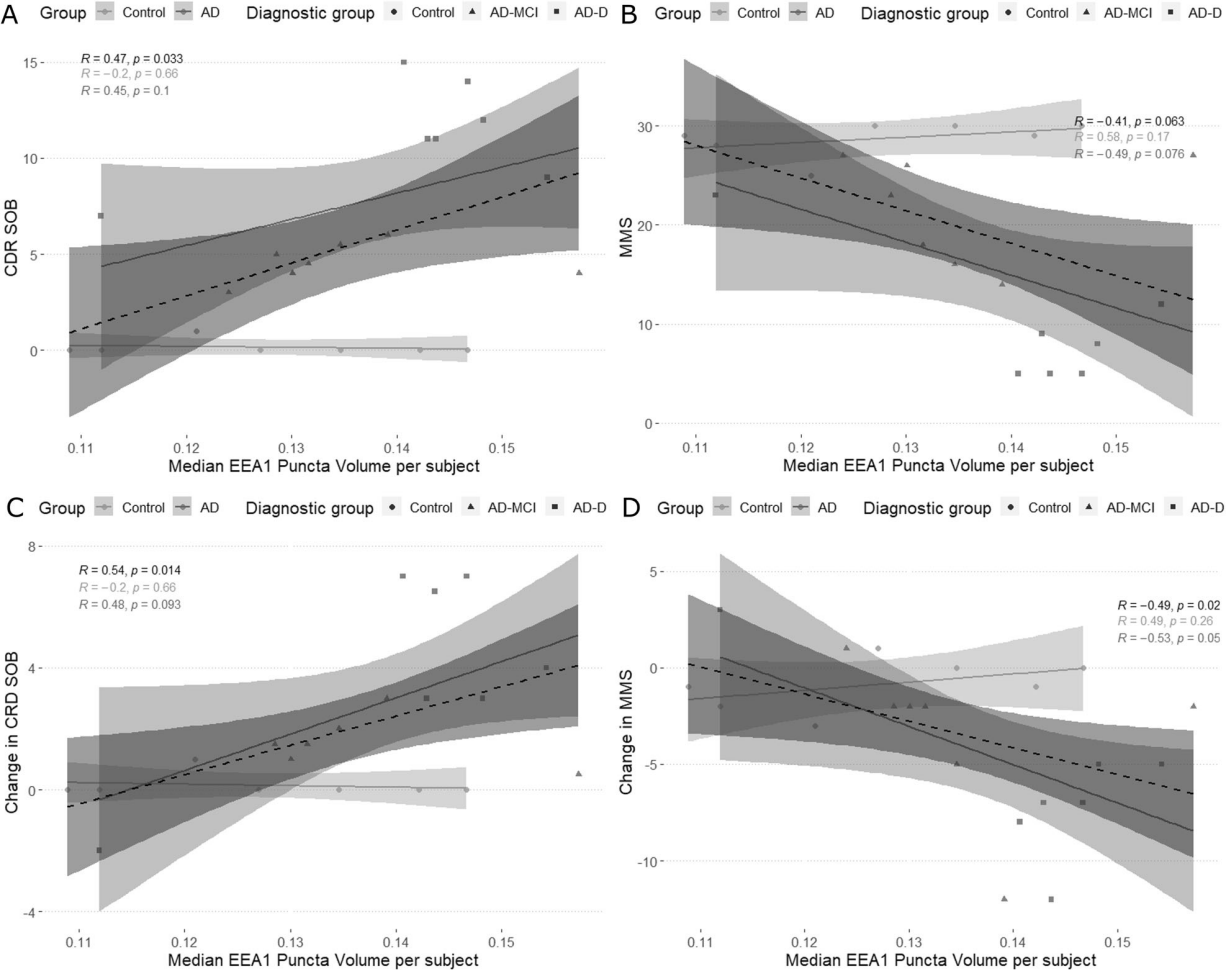
Graphical representation of the correlations between cognitive measures and the median EEA1 positive puncta volume. For all cases, circles represent controls, triangles represent AD-MCI, and squares represent AD-D. Likewise, the total correlation is represented as a dashed line in dark-grey and its 95% confidence interval is represented in the shaded dark grey area. The partial correlations according to disease group are in grey (AD) and light grey (Control). Correlation values are on the graph, with the total correlation being in black, the correlation for controls in light grey, and the correlations for AD in dark grey. Correlations between Median EEA1 puncta volume and **A** CDR Sum of Boxes, **B** Mini Mental State, **C** changes in CDR Sum of Boxes, and **D** changes in Mini Mental State.

Since we observed correlations between the volume of EEA1 positive puncta in fibroblasts of AD patients and amyloid deposition in the brain, we then evaluated whether the median EEA1 positive puncta volume correlated with cognitive measures. In this case, we used the cognitive measures taken the closest to the date of the skin biopsy as well as the changes in those values between the first visit and the biopsy visit. For the CDR, instead of using standardised values, we used the Sum of Boxes (SOB) which has a wider distribution and therefore is more adequate for correlations. Total correlations showed significance between CDR SOB (Fig. 5A and Supplementary Table 2) and its changes over time (Fig. 5C and Supplementary Table) and median EEA1 positive puncta volume, and an inverse correlation with the MMSE (Fig. 5B and Supplementary Table) and its changes over time (Fig. 4D and Supplementary Table). These correlations, however, could be driven by the floor and ceiling effects observed in controls, as controls had lower CDR SOB and higher MMSE scores. Stratified correlations for the AD group, showed that only the correlation between changes in MMSE and median volume of the EEA1 positive puncta remained significant (Fig. 5D and Supplementary Table). These correlations did not withstand multiple testing. Nevertheless, the trends observed for the rest of the variables are interesting as they link a worse cognitive performance and a higher deterioration of the cognitive function with the median volume of EEA1 positive puncta.

### Associations of median puncta volume with SNPs related to endosomal function

We then asked whether the endosomal alterations detected in fibroblasts of AD patients could be associated with genetic risk factors for AD. We thus calculated associations between SNPs recently identified in AD [25] and the volume of EEA1 positive puncta for each individual from the IMABio3 cohort studied here. We selected all SNPs that had a MAF > 0.01 in the general population and a MAF > 0 in our cohort. This selection left us with 74 SNPs (Supplementary Table 3). Our analyses were limited due to the sample size and the reported results are not corrected for multiple comparisons. Nevertheless, we found a significant association for three genes. Models were corrected for AD status. Homozygotes of the risk allele (C) for rs623742257 (*COX7C* gene) had larger EEA1 puncta volume than homozygotes for the T allele (estimated AMD = 0.023, *p* = 0.03) (Supplementary Fig. 1A). Another significant association was found for carriers of the G allele (risk allele) for rs1800978 (*ABCA1*), with larger EEA1 puncta volume than C homozygotes (estimated AMD = 0.0124, *p* = 0.026) (Supplementary Fig. 1B). The rs2242595 SNP of the *MYO15A* gene also showed association between homozygotes of the G allele (risk) and larger endosomal puncta than heterozygotes (estimated AMD = 0.019, *p* = 0.036) (Supplementary Fig. 1C). Non-significant trends were found for rs4985556 (*IL34*), with A allele carriers having larger EEA1 puncta (estimated AMD = 0.015, *p* = 0.053) (Supplementary Fig. 1D).

## Discussion

In this study, we have observed that EEA1 positive puncta in fibroblasts have increased volume in AD-D subjects compared to controls using confocal microscopy. Moreover, change in the morphology of the endosomal compartment was confirmed at the ultrastructural level. EM study suggests an increase in the number of endosomes rather than in their size leading to increase of endosomal area, as we demonstrated previously in fibroblasts from individuals with DS [17]. Although here we did not perform high pressure freezing EM without aldehyde fixation that prevents clustering of early endosomes as shown previously [17], we clearly identified in AD-MCI and AD-D clustering of early endosomes on EM pictures following aldehyde fixation that was not that visible in controls (Fig. 3). This clustering could be due to the higher number of endosomes as seen in fibroblasts in DS condition [17]. Nevertheless, increase of EEA1 positive puncta by immunofluorescence and confocal microscopy was correlated with several measures that have long been considered to be hallmarks of AD. On one hand, amyloid accumulation in the brain assessed by PET imaging using PiB retention, and, on the other hand, cognitive decline. Therefore, alterations of the endosomal compartment in peripheral cells have the potential to be used as proxy biomarkers of amyloid brain deposition (disease state) and cognition (disease stage).

Our data on the alteration of the endosomal compartment in fibroblasts of AD subjects confirm our previous observations in peripheral cells of AD patients [18] and in individuals with DS [17], and by others in DS [15]. Altogether, these results suggest a general dysregulation of the endo-lysosomal pathway that is not limited to the central nervous system. The question remains as whether these alterations are due to genetic factors, epigenetic changes, or circulating factors. It is known that sporadic AD has a strong genetic component explained by the accumulation of multiple genetic risk factors [32]. Indeed, we identified 3 SNPs that were associated with EAA1 puncta volume, with the risk allele for AD being associated with larger volume, indicating that several genes associated with AD could be increasing risk through altering the endo-lysosomal pathway. These three SNPs map to genes encoding *ABCA1*, *COX7C* and *MYO15A*. *ABCA1*, a transporter of cholesterol, plays a critical role in the lipidation of extracellular apolipoprotein and modulates late endocytic trafficking [33]. *COX7A* is part of the mitochondrial genes that were found altered in blood early in AD [34]. *MYO15A* is a myosin involved in actin organisation in mechanosensory hair bundles [35]. Although no direct link with the endosomal compartment has been found yet, the role of actin in retromer function is well described and retromer dysfunction is occurring in neurodegenerative disease including AD [36].

Alternatively, peripheral endosomal alterations could be due to circulating molecules, such as amyloid peptides or tau. Indeed, glial cells treated with Aβ_1–42_ peptides show alterations in their endo-lysosomal pathway [37]. We could not detect sufficient amounts of secreted Aβ peptides, either 38, 40 or 42 using the MesoScale Discovery platform by fibroblasts from controls, AD-D and AD-MCI subjects. In addition, since fibroblasts have been cultured for several passages, it is unlikely that circulating molecules, even if accumulated in the cells, would still be remaining. Therefore, it is possible that circulating molecules or environmental factors could elicit epigenetic changes that would translate into a dysregulation of the endosomal compartment. Modification of the histone deacetylase activity in APOE4 astrocytes was shown to revert the endosomal phenotype [38], thus suggesting that epigenetic modifications lead to alterations of the endo-lysosomal pathway. Further experiments are needed to fully understand the origin of this alteration in peripheral cells.

Correlations between PiB and EEA1 positive puncta volume in PBMCs had already been described in this cohort [18], although the subjects used for the two studies barely overlap. Although our correlations did not reach significance after correction for multiple comparison, the uncorrected results deserve discussion. As in this previous study, we observed a correlation between the volume of EEA1 positive puncta and global brain PiB retention, as well as with PiB retention in the anterior and middle cingulum. Additional trends for correlation could be observed for the AD group in the posterior cingulum and precuneus. Despite these correlations not being significant, they could have a biological relevance, as there is a strong relationship between the endo-lysosomal compartment and the amyloidogenic metabolism. Likewise, correlations with cognitive variables, including worsening of the symptoms between the first visit and the biopsy, although non-significant after correction, were observed. At time of biopsy, the volume of the puncta correlated positively with CDR SOB and negatively with MMSE, therefore subjects with larger EEA1 positive puncta volume had worse cognitive performance. However, since controls have both low scores of CDR SOB and high scores of MMSE, total correlations are biased. When performing stratified correlations, those were only a trend. Similarly, subjects showing the highest changes both in CDR SOB and MMSE between baseline and biopsy had largest EEA1 positive puncta volume. Although correlations with PiB and cognition are non-significant, they all went in a similar direction and confirmed previous correlations obtained in PBMCs [18]. Therefore, it could be interesting to further study the correlations between EEA1 positive puncta volume and cognitive and neuroimaging markers, especially its potential predictive power on amyloid and tau pathology and cognitive decline. No significant correlations could be observed between EEA1 positive puncta volume and CSF biomarkers nor MRI measures.

Although our conclusions are limited due to the sample size, we need to consider the difficulties to obtain skin biopsies, as not all participants of the IMABio3 cohort agreed to undergo the procedure. Other studies using fibroblasts of AD [18] or DS [17] patients had much smaller sample sizes, even studies analysing *post-mortem* brain tissue were limited in sample size [6, 15]. Therefore, despite these limitations, our study is in line with previous analyses, and it is strengthened by the fact that we analysed a high number of cells (75) for each of the included participants. The availability of samples limited the possibility of analysing the effect of APOE on endosomal size. Indeed, all controls were *APOEε4* non-carriers and we would not be able to discern between disease and genotype effect. There is literature pointing out to the effect of *APOEε4* on the expression of genes linked to the endo-lysosomal pathway which translated into an increase in Rab5 positive endosome numbers [39]. In addition, because the samples available for analysis were not well matched in terms of sex, we could not add sex as confounder and further studies will be needed to fully assess the effect of sex on endosomal morphology. Another limitation of our study is the delay between the PiB acquisition and the biopsy, as they were not performed at the same time. In addition, the exploration of the endosomal phenotypes caused by genetic factors, especially in iPSC-derived cells, could explain how some of these are linked to AD risk, as enlarged endosomal puncta have been observed in neurons derived from iPSC from sporadic AD patients [40] and from an individual with DS [17].

In conclusion, we observed enlarged EEA1 positive puncta volume, a hallmark of endosomal alterations, in AD patients, particularly at the dementia stage of the disease, that tend to correlate with amyloid load and cognition, therefore showing potential as biomarkers for AD. The fact that EEA1 positive puncta volume also correlates to changes in cognition during the time interval preceding the skin biopsy reveals potential utility to predict cognitive decline that will need to be further validated in prospective cohorts.

## Supplementary information

Supplementary Table 1

Supplementary Table 2

Supplementary Table 3

Supplementary Figure 1

Supplementary Figure 1caption
