# Supplementary material for: Modifications of the endosomal compartment in fibroblasts from sporadic Alzheimer’s disease patients are associated with cognitive impairment
Source: Transl Psychiatry. 2023 Feb 14;13:54. doi: 10.1038/s41398-023-02355-z (PMC9929231; doi:10.1038/s41398-023-02355-z)
Supplement: Supplementary file 1 — Supplementary Table 1 [file 41398_2023_2355_MOESM1_ESM.docx]

Supplementary Table 1 : Correlations between PiB retention and EEA1 positive puncta volume. Correlations were total or stratified (AD and Controls). P-values were adjusted using false discovery rate.

|  |  | **Total**  **N=21** | **AD**  **N=14** | **Controls**  **N=7** |
| --- | --- | --- | --- | --- |
| **PiB GCI** | rho | 0.4 | 0.51 | -0.18 |
|  | p | 0.07 | 0.067 | 0.71 |
|  | p. adj. | 0.26 | 0.26 | 0.71 |
| **Frontal CTX** | rho | 0.32 | 0.4 | -0.18 |
|  | p | 0.15 | 0.16 | 0.71 |
|  | p.adj | 0.26 | 0.26 | 0.71 |
| **Ant Cing** | rho | 0.45 | 0.51 | -0.18 |
|  | p | 0.038 | 0.06 | 0.71 |
|  | p.adj | 0.26 | 0.26 | 0.71 |
| **Med Cing** | rho | 0.38 | 0.49 | -0.18 |
|  | p | 0.091 | 0.078 | 0.71 |
|  | p.adj | 0.26 | 0.26 | 0.71 |
| **Post Cing** | rho | 0.32 | 0.5 | -0.5 |
|  | p | 0.15 | 0.072 | 0.27 |
|  | p.adj | 0.26 | 0.26 | 0.38 |
| **Precuneus** | rho | 0.33 | 0.49 | -0.32 |
|  | p | 0.14 | 0.081 | 0.5 |
|  | p.adj | 0.26 | 0.26 | 0.63 |
| **Pariet CTX** | rho | 0.31 | 0.44 | -0.32 |
|  | p | 0.18 | 0.12 | 0.5 |
|  | p.adj | 0.27 | 0.26 | 0.63 |
| **Temp CTX** | rho | 0.32 | 0.45 | -0.18 |
|  | p | 0.16 | 0.11 | 0.71 |
|  | p.adj | 0.26 | 0.26 | 0.71 |
