# Supplementary material for: Modifications of the endosomal compartment in fibroblasts from sporadic Alzheimer’s disease patients are associated with cognitive impairment
Source: Transl Psychiatry. 2023 Feb 14;13:54. doi: 10.1038/s41398-023-02355-z (PMC9929231; doi:10.1038/s41398-023-02355-z)
Supplement: Supplementary file 2 — Supplementary Table 2 [file 41398_2023_2355_MOESM2_ESM.docx]

Supplementary Table 2 : Correlations between cognitive measures retention and EEA1 positive puncta volume. Correlations were total or stratified (AD and Controls). P-values were adjusted using false discovery rate.

|  |  | **Total**  **N=21** | **AD**  **N=14** | **Controls**  **N=7** |
| --- | --- | --- | --- | --- |
| **CDR SOB** | rho | 0.466 | 0.453 | -0.204 |
|  | p | 0.033 | 0.103 | 0.66 |
|  | p. adj. | 0.13 | 0.15 | 0.66 |
| **MMSE** | rho | -0.413 | -0.489 | 0.580 |
|  | p | 0.063 | 0.076 | 0.17 |
|  | p.adj | 0.15 | 0.15 | 0.23 |
| **Δ CDR SOB** | rho | 0.542 | 0.485 | -0.204 |
|  | p | 0.014 | 0.093 | 0.66 |
|  | p.adj | 0.13 | 0.15 | 0.66 |
| **Δ MMSE** | rho | -0.488 | -0.528 | 0.491 |
|  | p | 0.025 | 0.052 | 0.263 |
|  | p.adj | 0.13 | 0.15 | 0.32 |
