# Supplementary material for: Modifications of the endosomal compartment in fibroblasts from sporadic Alzheimer’s disease patients are associated with cognitive impairment
Source: Transl Psychiatry. 2023 Feb 14;13:54. doi: 10.1038/s41398-023-02355-z (PMC9929231; doi:10.1038/s41398-023-02355-z)
Supplement: Supplementary file 3 — Supplementary Table 3 [file 41398_2023_2355_MOESM3_ESM.docx]

Supplementary Table 3: List of the analyzed SNPs, adapted from Bellenguez et al 2022. Table contains chromosome and position information for each SNP, as well as gene and minor/major alleles. Minor allele frequencies (MAF) and Odds Ratio (OR) are those reported on the paper. OR represents risk of AD for the minor allele.

| Variant | Chromosome | Position | Gene | Known locus | Minor/major allele | MAF | OR |
| --- | --- | --- | --- | --- | --- | --- | --- |
| rs679515 | 1 | 207577223 | CR1 | CR1 | T/C | 0.188 | 1.13 |
| rs72777026 | 2 | 9558882 | ADAM17 |  | G/A | 0.144 | 1.06 |
| rs17020490 | 2 | 37304796 | PRKD3 |  | C/T | 0.145 | 1.06 |
| rs6733839 | 2 | 127135234 | BIN1 | BIN1 | T/C | 0.389 | 1.17 |
| rs139643391 | 2 | 202878716 | WDR12 |  | T/TC | 0.131 | 0.94 |
| rs10933431 | 2 | 233117202 | INPP5D | INPP5D | G/C | 0.234 | 0.93 |
| rs16824536 | 3 | 155069722 | MME |  | A/G | 0.054 | 0.92 |
| rs3822030 | 4 | 993555 | IDUA |  | G/T | 0.429 | 0.95 |
| rs6846529 | 4 | 11023507 | CLNK | CLNK/HS3ST1 | C/T | 0.283 | 1.07 |
| rs2245466 | 4 | 40197226 | RHOH |  | G/C | 0.343 | 1.05 |
| rs112403360 | 5 | 14724304 | ANKH |  | A/T | 0.073 | 1.09 |
| rs62374257 | 5 | 86927378 | COX7C |  | C/T | 0.23 | 1.07 |
| rs871269 | 5 | 151052827 | TNIP1 |  | T/C | 0.326 | 0.96 |
| rs113706587 | 5 | 180201150 | RASGEF1C |  | A/G | 0.11 | 1.09 |
| rs6605556 | 6 | 32615322 | HLA-DQA1 | HLA | G/A | 0.161 | 0.91 |
| rs10947943 | 6 | 41036354 | UNC5CL | TREM2 | A/G | 0.142 | 0.94 |
| rs60755019 | 6 | 41181270 | TREML2 | TREM2 | G/A | 0.004 | 1.55 |
| rs7767350 | 6 | 47517390 | CD2AP | CD2AP | T/C | 0.271 | 1.08 |
| rs785129 | 6 | 114291731 | HS3ST5 |  | T/C | 0.35 | 1.04 |
| rs6943429 | 7 | 7817263 | UMAD1 |  | T/C | 0.42 | 1.05 |
| rs10952097 | 7 | 8204382 | ICA1 |  | T/C | 0.114 | 1.07 |
| rs13237518 | 7 | 12229967 | TMEM106B |  | A/C | 0.411 | 0.96 |
| rs1160871 | 7 | 28129126 | JAZF1 |  | G/GTCTT | 0.222 | 0.95 |
| rs6966331 | 7 | 37844191 | EPDR1 | NME8 | T/C | 0.349 | 0.96 |
| rs76928645 | 7 | 54873635 | SEC61G |  | T/C | 0.103 | 0.93 |
| rs7384878 | 7 | 100334426 | SPDYE3 | ZCWPW1/NYAP1 | C/T | 0.31 | 0.92 |
| rs11771145 | 7 | 143413669 | EPHA1 | EPHA1 | A/G | 0.348 | 0.95 |
| rs1065712 | 8 | 11844613 | CTSB |  | C/G | 0.053 | 1.09 |
| rs73223431 | 8 | 27362470 | PTK2B | PTK2B | T/C | 0.369 | 1.07 |
| rs11787077 | 8 | 27607795 | CLU | CLU | T/C | 0.392 | 0.91 |
| rs34173062 | 8 | 144103704 | SHARPIN |  | A/G | 0.081 | 1.13 |
| rs1800978 | 9 | 104903697 | ABCA1 |  | G/C | 0.13 | 1.06 |
| rs7912495 | 10 | 11676714 | USP6NL | ECHDC3 | G/A | 0.462 | 1.06 |
| rs7068231 | 10 | 60025170 | ANK3 |  | T/G | 0.403 | 0.95 |
| rs6586028 | 10 | 80494228 | TSPAN14 |  | C/T | 0.196 | 0.93 |
| rs6584063 | 10 | 96266650 | BLNK |  | G/A | 0.043 | 0.89 |
| rs7908662 | 10 | 122413396 | PLEKHA1 |  | G/A | 0.467 | 0.96 |
| rs10437655 | 11 | 47370397 | SPI1 | CELF1/SPI1 | A/G | 0.399 | 1.06 |
| rs1582763 | 11 | 60254475 | MS4A4A | MS4A | A/G | 0.371 | 0.91 |
| rs3851179 | 11 | 86157598 | EED | PICALM | T/C | 0.358 | 0.9 |
| rs74685827 | 11 | 121482368 | SORL1 | SORL1 | G/T | 0.019 | 1.19 |
| rs11218343 | 11 | 121564878 | SORL1 | SORL1 | C/T | 0.039 | 0.84 |
| rs6489896 | 12 | 113281983 | TPCN1 |  | C/T | 0.076 | 1.08 |
| rs7401792 | 14 | 92464917 | SLC24A4 | SLC24A4/RIN3 | G/A | 0.371 | 1.04 |
| rs12590654 | 14 | 92472511 | SLC24A4 | SLC24A4/RIN3 | A/G | 0.328 | 0.93 |
| rs7157106 | 14 | 105761758 | IGH gene cluster |  | A/G | 0.36 | 1.05 |
| rs10131280 | 14 | 106665591 | IGH gene cluster |  | A/G | 0.133 | 0.94 |
| rs8025980 | 15 | 50701814 | SPPL2A | SPPL2A | G/A | 0.345 | 0.96 |
| rs602602 | 15 | 58764824 | MINDY2 | ADAM10 | A/T | 0.28 | 0.94 |
| rs117618017 | 15 | 63277703 | APH1B | APH1B | T/C | 0.144 | 1.11 |
| rs3848143 | 15 | 64131307 | SNX1 |  | G/A | 0.22 | 1.05 |
| rs12592898 | 15 | 78936857 | CTSH |  | A/G | 0.133 | 0.94 |
| rs1140239 | 16 | 30010081 | DOC2A |  | T/C | 0.379 | 0.94 |
| rs889555 | 16 | 31111250 | BCKDK | KAT8 | T/C | 0.281 | 0.95 |
| rs4985556 | 16 | 70660097 | IL34 | IL34 | A/C | 0.115 | 1.07 |
| rs450674 | 16 | 79574511 | MAF |  | C/T | 0.373 | 0.96 |
| rs12446759 | 16 | 81739398 | PLCG2 | PLCG2 | G/A | 0.403 | 0.95 |
| rs16941239 | 16 | 86420604 | FOXF1 |  | A/T | 0.029 | 1.13 |
| rs56407236 | 16 | 90103687 | PRDM7 |  | A/G | 0.069 | 1.11 |
| rs35048651 | 17 | 1728046 | WDR81 |  | T/TGAG | 0.214 | 1.06 |
| rs7225151 | 17 | 5233752 | SCIMP | SCIMP/RABEP1 | A/G | 0.124 | 1.08 |
| rs2242595 | 17 | 18156140 | MYO15A |  | A/G | 0.112 | 0.94 |
| rs5848 | 17 | 44352876 | GRN |  | T/C | 0.289 | 1.07 |
| rs199515 | 17 | 46779275 | WNT3 | MAPT | G/C | 0.219 | 0.94 |
| rs2526377 | 17 | 58332680 | TSPOAP1 | TSPOAP1 | G/A | 0.445 | 0.95 |
| rs4277405 | 17 | 63471557 | ACE | ACE | C/T | 0.384 | 0.94 |
| rs12151021 | 19 | 1050875 | ABCA7 | ABCA7 | A/G | 0.336 | 1.1 |
| rs149080927 | 19 | 1854254 | KLF16 |  | G/GC | 0.48 | 1.05 |
| rs9304690 | 19 | 49950060 | SIGLEC11 |  | T/C | 0.24 | 1.05 |
| rs587709 | 19 | 54267597 | LILRB2 |  | C/T | 0.325 | 1.05 |
| rs1358782 | 20 | 413334 | RBCK1 |  | A/G | 0.246 | 0.95 |
| rs6014724 | 20 | 56423488 | CASS4 | CASS4 | G/A | 0.09 | 0.89 |
| rs6742 | 20 | 63743088 | SLC2A4RG |  | T/C | 0.221 | 0.95 |
| rs2154481 | 21 | 26101558 | APP |  | C/T | 0.476 | 0.95 |
| rs2830489 | 21 | 26775872 | ADAMTS1 | ADAMTS1 | T/C | 0.281 | 0.95 |
