# Supplementary figures and images for: Modifications of the endosomal compartment in fibroblasts from sporadic Alzheimer’s disease patients are associated with cognitive impairment
Source: Transl Psychiatry. 2023 Feb 14;13:54. doi: 10.1038/s41398-023-02355-z (PMC9929231; doi:10.1038/s41398-023-02355-z)

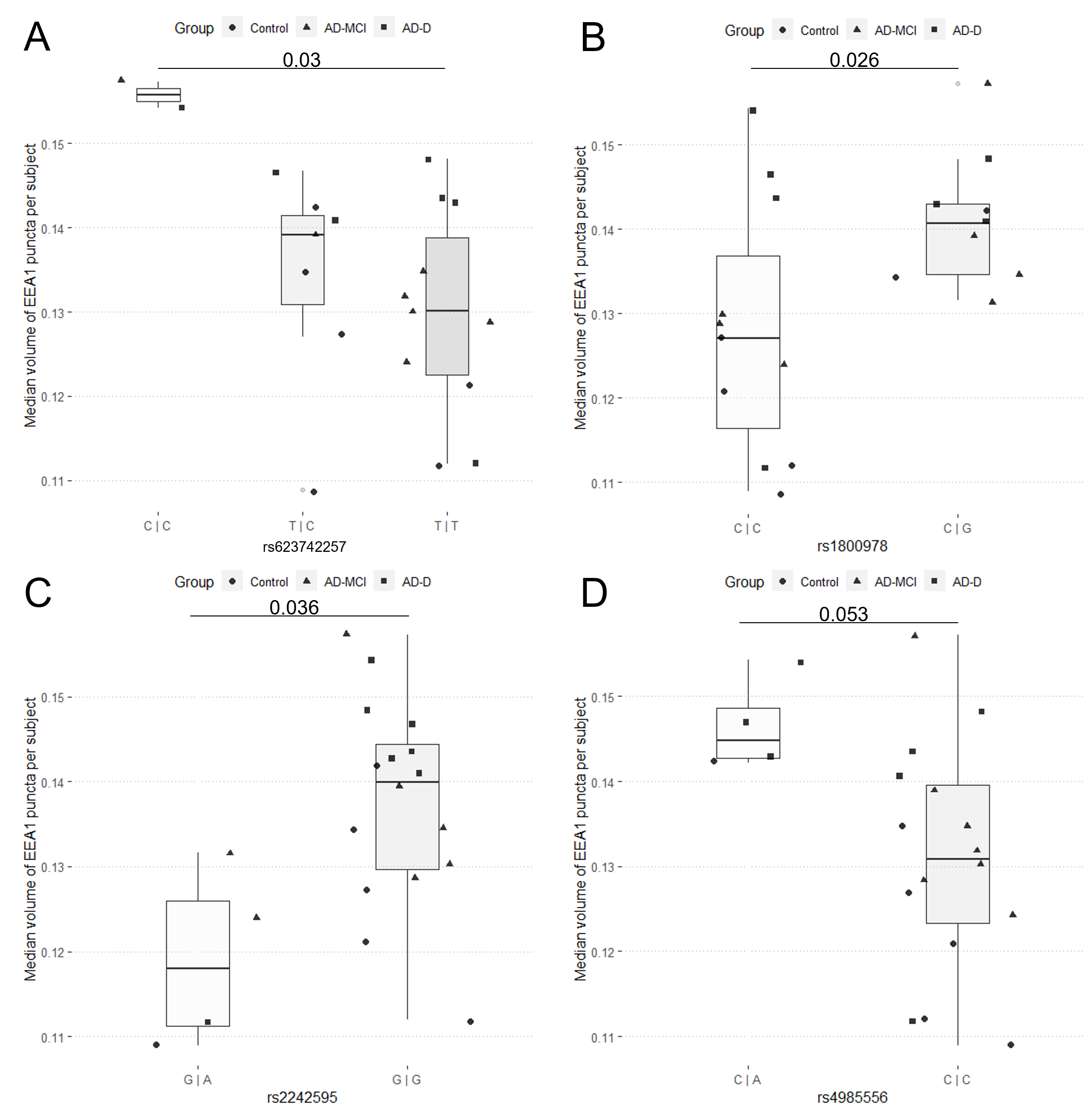

Supplement: Supplementary file 4 — Supplementary Figure 1 [file 41398_2023_2355_MOESM4_ESM.tif]
