# Supplementary material for: Modifications of the endosomal compartment in fibroblasts from sporadic Alzheimer’s disease patients are associated with cognitive impairment
Source: Transl Psychiatry. 2023 Feb 14;13:54. doi: 10.1038/s41398-023-02355-z (PMC9929231; doi:10.1038/s41398-023-02355-z)
Supplement: Supplementary file 5 — Supplementary Figure 1caption [file 41398_2023_2355_MOESM5_ESM.docx]

Supplementary Figure 1: Graphical representation of the differences in puncta volume according to genotype of the different SNPs: rs623742257 (COX7), rs1800978 (ABCA1), rs2242595 (MYO15A), and rs4985556 (IL34). Circles represent participants in the Control group, triangles those in the AD-MCI group, and squares those in the AD-D group.
